# Asymmetric Schottky Contacts in van der Waals Metal-Semiconductor-Metal Structures Based on Two-Dimensional Janus Materials

**DOI:** 10.34133/2020/6727524

**Published:** 2020-11-15

**Authors:** Jia Liu, Ji-Chang Ren, Tao Shen, Xinyi Liu, Christopher J. Butch, Shuang Li, Wei Liu

**Affiliations:** ^1^Nano and Heterogeneous Materials Center, School of Materials Science and Engineering, Nanjing University of Science and Technology, Nanjing 210094, China; ^2^Department of Biomedical Engineering, Nanjing University, Nanjing, China; ^3^Blue Marble Space Institute of Science, Seattle, Washington, USA

## Abstract

Physical and electronic asymmetry plays a crucial role in rectifiers and other devices with a directionally variant current-voltage (*I-V*) ratio. Several strategies for practically creating asymmetry in nanoscale components have been demonstrated, but complex fabrication procedures, high cost, and incomplete mechanistic understanding have significantly limited large-scale applications of these components. In this work, we present density functional theory calculations which demonstrate asymmetric electronic properties in a metal-semiconductor-metal (MSM) interface composed of stacked van der Waals (vdW) heterostructures. Janus MoSSe has an intrinsic dipole due to its asymmetric structure and, consequently, can act as either an n-type or p-type diode depending on the face at the interior of the stacked structure (SeMoS-SMoS vs. SMoSe-SMoS). In each configuration, vdW forces dominate the interfacial interactions, and thus, Fermi level pinning is largely suppressed. Our transport calculations show that not only does the intrinsic dipole cause asymmetric *I-V* characteristics in the MSM structure but also that different transmission mechanisms are involved across the S-S (direct tunneling) and S-Se interface (thermionic excitation). This work illustrates a simple and practical method to introduce asymmetric Schottky barriers into an MSM structure and provides a conceptual framework which can be extended to other 2D Janus semiconductors.

## 1. Introduction

Physical and electronic asymmetry plays a crucial role in devices like rectifiers [1–4], which produce current-voltage (*I-V*) outputs and vary directionally depending on the applied bias. At the nanoscale, one strategy to produce current asymmetry is through metal-semiconductor-metal (MSM) structures composed of two metal-semiconductor (MS) junctions with distinct Schottky barriers connected back to back [5, 6]. In such an MSM, when a nonzero bias voltage is applied, one Schottky barrier is forward biased and the other one is reversed biased [6]. Compared to a single Schottky barrier MS diode, the second MS interface of the MSM diode further modulates current allowing backward current to also vary with voltage. An asymmetric MSM structure can be created by several strategies including using two electrodes with different work functions [7], asymmetric contact geometries [8], externally applied mechanical forces (*e.g*., piezoelectric-potential-controlled Schottky diodes) [5], and enhanced metal oxidation or defect density differences [9]. However, large-scale application of these techniques is limited by high cost and difficulties in fabrication which include force-induced instability, shape selection, and Fermi level pinning (FLP) [8, 10]. Among these, FLP caused by defects is the most universal [7], can significantly hinder effective control of Schottky barriers at the contact, and requires complicated treatment of the bulk metal to avoid [11]. To overcome these obstacles, herein, we propose a new class of MSM structure fabricated by stacking two-dimensional (2D) Janus materials and 2D metals. Compared to other approaches, fabrication of these MSMs is straightforward and effective since (1) the surface of these 2D materials is well controlled; (2) the dipole which generates the surface asymmetry is inherent to the Janus material (*e.g*., MoSSe [12] and GeSe [13]); (3) the interface of the layered 2D materials is dominated by van der Waals (vdW) forces rather than chemical bonds, significantly suppressing the effect of FLP; and (4) asymmetric Schottky contacts can be created without requiring external forces, shape selection, doping, or precise control of contact geometries.

The term “Janus” is derived from the two-faced Roman god of transitions, and in nanotechnology, it refers to structures with two distinct faces, usually due to different terminating atoms on opposite surfaces [14, 15]. A large variety of 2D Janus materials have been predicted theoretically and/or fabricated experimentally [16–20]. Examples include Janus transition-metal dichalcogenides (TMDCs, such as MoSSe, WSSe, MoSTe, and WSTe) [16, 19, 21–29], Janus metal-nitrides and metal-carbides (MXenes, such as Cr_2_COF and V_2_COF) [14], Janus graphene [15, 18, 30], Janus black arsenic-phosphorus [31], Janus group-III chalcogenides (such as Ga_2_SSe, In_2_SSe, Ga_2_STe, and In_2_STe) [19, 32], and Janus group IV monochalcogenides (such as GeSe and SnS) [13, 33]. These asymmetric structures generate intrinsic electric fields (*F*) associated with the interior dipole across the plane, causing the two surfaces to have different work functions, and consequently, different Schottky barriers are generated dependent on the interfacial surface. On the basis of these observations, we hypothesized that when a Janus monolayer, such as MoSSe, is used as the semiconductor in MSM structures, the intrinsic dipole may introduce electronic asymmetry in a simple and practical manner.

In this contribution, we propose an asymmetric MSM design composed of layered Janus MoSSe and 1T-phase MoS_2_. MoSSe has been fabricated experimentally, by substituting S (Se) atoms with Se (S) in the exposed surface of a MoS_2_ (MoSe_2_) monolayer [16, 22]. The metallic 1T-phase MoS_2_ [34] was selected to avoid FLP problems associated with the use of bulk metals as contacts [7, 35, 36]. While metallic 1T MoS_2_ [34] is dynamically instable, it has been demonstrated experimentally [37] and was selected, largely, as a prototype to demonstrate that FLP problems associated with the use of bulk metals as contacts can be avoided through employing 2D materials [7, 35, 36]. Our calculations predict different types of Schottky contacts at the interface of MoSSe/MoS_2_ heterostructures: the n-type S-S interface and p-type S-Se interface. In both cases, the intrinsic dipole of MoSSe is preserved, while the FLP effects are significantly suppressed as the 2D materials are fully bonded and interact through van der Waals (vdW) forces [38, 39]. In this MSM structure, the asymmetric Schottky barriers originate from the reversal of the inherent electric field in Janus MoSSe, which always points from Se to S atoms. The carrier transports under positive and negative bias voltages are found to be dominated by thermionic excitation and tunneling, respectively, further underscoring the asymmetry of the structure. Although discussions of our findings are mainly based on MoSSe, the concepts that we outline here can be extended to other 2D Janus semiconductors.

## 2. Computational Methods

Geometry optimizations and electronic property calculations were performed using the Vienna Ab initio Simulation Package (VASP) [40, 41]. The generalized gradient approximation (GGA) with Perdew-Burke-Ernzerhof (PBE) algorithm [42] was selected as the exchange-correlation functional. The interaction between ionic core and valance electrons was described using the projector-augmented wave (PAW) method [43, 44]. The DFT+vdW method of Tkatchenko and Scheffler [45] was employed to account for long-range vdW interactions. The DFT+vdW method was constructed from mean-field electronic structure calculations and has been shown to perform well in describing vdW forces between 2D materials [46, 47]. An energy cutoff of 400 eV and a Monkhorst-Pack scheme with a *k*-point mesh of 9 × 9 × 1 were employed to ensure accuracy. Structures were relaxed with energy change converged to 1 × 10^−5^ eV/cell in electronic self-consistency cycles and all forces smaller than 0.01 eV/Å in ionic relation loops. The vacuum width is larger than 15 Å in the *z* direction to avoid interaction between periodic slabs. When calculating the Schottky barriers *ϕ*_p_ and *ϕ*_n_, the *z* axis is set to 150 Å. The *I-V* characteristics and other transport properties of the contacts were calculated using the nonequilibrium Green's function (NEGF) method within the frame of DFT, as implemented in the Atomistix ToolKit (ATK) package [48]. We used numerical linear combination of atomic orbitals (LCAO) basis sets in device calculations.

The optimized lattice constants *a* for Janus MoSSe and 1T MoS_2_ are 3.23 and 3.13 Å, respectively, consistent with previous studies [16, 49, 50]. The Mo-S bond distance in 1T MoS_2_ is 2.43 Å. In MoSSe, the bond distance of Mo-S slightly decreases to 2.41 Å while Mo-Se is 2.53 Å due to the larger radius of the Se atom. After stacking, the relaxed lattice constants, *a*_A_ and *a*_B_, of contacts with the S‐S(*C*_S−S_)andS‐Se(*C*_S‐Se_) interfaces are 3.21 and 3.20 Å, respectively, with a lattice mismatch (*a*_MoS2‐_*a*_MoSSe_)/*a*_MoS2_ = 3.19%.

## 3. Results and Discussion

**Figure 1:**
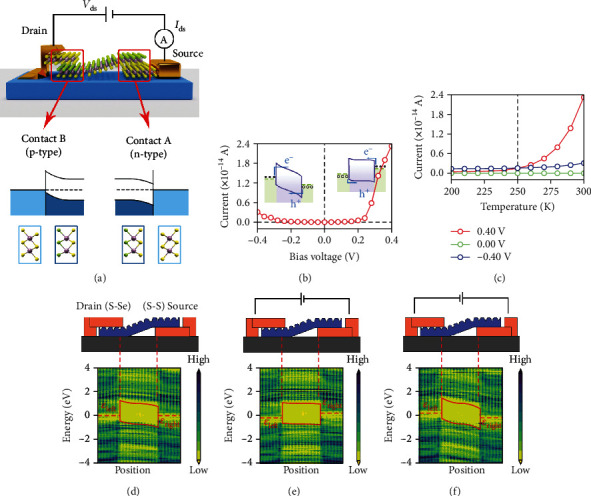
(a) Scheme of asymmetric van der Waals metal-semiconductor-metal diode based on two-dimensional Janus MoSSe (scattering region) and 1T MoS_2_ (drain and source electrodes). The red squares indicate different contact interfaces with disparate Schottky barrier heights and band bending directions (S-S contact at source side with an n-type barrier and S-Se contact at drain side with a p-type barrier, marked as *C*_S−S_ and *C*_S−Se_, respectively). The violet, yellow, and green spheres represent Mo, S, and Se atoms, respectively. (b) The corresponding *I-V* characteristic curve at 300 K of this device. The illustrations are schematic band diagrams under negative (left) and positive (right) bias voltages, respectively. The purple curves represent the boundary of electrodes and bending of CB and VB. The blue arrows represent the primary transferring methods through contacts (tunneling and thermionic excitation under negative and positive voltages, respectively). The green dashed lines indicate the Fermi levels in two electrodes. The hollow and stuffed circles denote holes and electrons, respectively. (c) The current versus temperature from 200 to 300 K of the MSM structure under different bias voltages. The vertical dashed line represents the critical temperature where red and blue curves are crossed around 250 K. (d)-(f) The spatially resolved local density of states (LDOS) projected on the position of contact under *V*_ds_ = 0.00, 0.40, and −0.40 V. The horizontal red dashed lines represent the chemical potentials (*ε*) of electrodes. The red curves illustrate the boundaries of electrodes and theoretical band diagrams. The device configurations are depicted above LDOS graphs for references. The orange squares represent 1T MoS_2_, while the blue layer with one side flat and the other side fluctuant represents the Janus MoSSe semiconductor. The “S-S” and “S-Se” tags indicate *C*_S−S_ and *C*_S−Se_, respectively.

Our calculations show that MoSSe is a semiconductor with a direct band gap of 1.63 eV, consistent with the experimental optical gap (1.68 eV) [22], while 1T MoS_2_ is a 2D metal (see Figure S1). As shown in Figure 1(a), the asymmetric MSM structure has two contacts, where both contacts *C*_S−S_ (right side) and *C*_S‐Se_ (left side) are vdW heterojunctions. Since *C*_S−S_ and *C*_S‐Se_ exhibit different Schottky barrier heights (SBHs), this sandwich structure yields an asymmetric *I-V* characteristic.  Figure 1(b) shows that the forward current from drain to source *I*_ds_(2.34 × 10^−14^Aunder0.40Vand300K) is one order of magnitude larger than the backward *I*_ds_(3.10 × 10^−15^Aunder0.40Vand300K). Under positive *V*_ds_, the electrons drift from source to drain through the n-type barrier of *C*_S−S_, while the holes drift in the opposite direction and across the p-type barrier of *C*_S‐Se_. Under negative *V*_ds_, barriers for both electrons and holes increase relative to under positive *V*_ds_, as shown in the schematic band diagrams in Figure 1(b).

Figure 1(c) shows that under negative *V*_ds_, the *I*_ds_ varies slightly with increasing temperature (*T*). Under positive bias, *I*_ds_ has a much greater sensitivity to temperature. These results suggest different transport mechanisms between the two directions. To discern the transport mechanisms contributing to the current under each condition, we plotted the spatially resolved local density of states (LDOS) along the transport direction under *V*_ds_ = 0and ± 0.40V (Figure 1(d)–1(f)). The flat conduction band (CB) and valence band (VB) at the metal-semiconductor interface (right illustration in Figures 1(b) and 1(e)), along with the exponential *I-V* curve (Figure 1(b)), indicate that carrier transport under positive bias voltages is mainly through thermionic excitation [51, 52]. In contrast, the CB and VB near the interface bend downward with the barrier becoming sharp under negative *V*_ds_ (Figure 1(b) left, and Figure 1(f)) leading to *I*_ds_which appears to increase quadratically relative to *V*_ds_ (Figure 1(b)). These results illustrate that carrier transport in this case is consistent with the Fowler-Nordheim (F-N) model [53], implying it occurs mainly through a tunneling mechanism. In the F-N model, $I\left(V\right)\propto V^{2}\exp\left(-4d_{\phi }\sqrt{2m^{*}\phi^{3}}/\left(3\hbar qV\right)\right)$ [53, 54], where *ħ* is the reduced Planck constant, *m*^∗^ is the effective mass of carrier in system, and *d*_*ϕ*_ is the barrier width. These trends show that forward conduction in the MSM occurs through thermionic excitation while back-current occurs through F-N tunneling. Due to these different mechanisms, the scaling of these effects with temperature varies, yielding a temperature critical point (*T*_0_) around 250 K where the preferred direction of conduction inverts at *T*_0_ (when *T* < *T*_0_, *I*_ds_(0.40V) < *I*_ds_(−0.40V), while it reversed when *T* > *T*_0_). Consequently, the temperature dependence of thermionic transport has a key influence on the rectification properties of the MSM.

**Figure 2:**
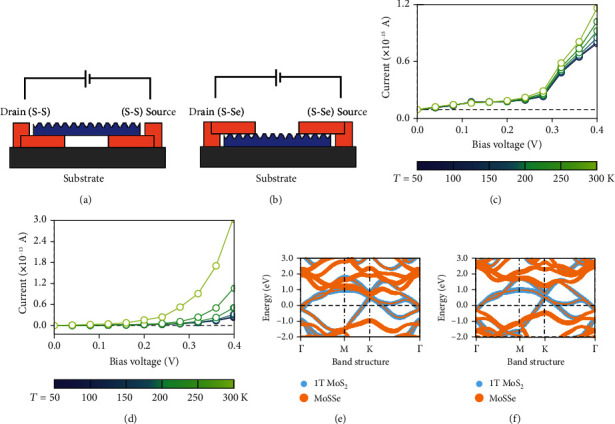
(a), (b) The symmetric devices made from *C*_S−S_ and *C*_S−Se_ and marked as *D*_S−S_ and *D*_S−Se_, respectively. (c), (d) The *I-V* characteristic curves under different temperatures of *D*_S−S_ and *D*_S−Se_, respectively. The electrode temperatures are ranged from 50 to 300 K. The bias voltages are ranged from 0.00 to 0.40 V because the *I-V* curves under positive and negative bias are symmetric. Note that the current in two devices is in different orders of magnitudes due to disparate transmission mechanisms. (e), (f) The projected band structures of *C*_S−S_ and *C*_S−Se_, respectively. The sizes of the blue and orange dots represent the relative weights of 1T MoS_2_ and Janus MoSSe layers, respectively.

Given that each MS junction contributes to the overall device performance, we constructed two conventional symmetric MSM structures composed of two of each interface (S-S in Figure 2(a) and S-Se in Figure 2(b)) for comparison to the asymmetric device discussed above. Like the asymmetric MSM structure, the electrode and central region lengths for these devices were set to 5.55 and 66.64 Å, respectively, for *D*_S‐S_ (*i.e*., symmetric device made from two *C*_S‐S_) and 5.55 and 66.57 Å, respectively, for *D*_S‐Se_ (device with two *C*_S‐Se_). As expected, the computed *I-V* curves exhibit a diode-like feature for both *D*_S‐S_ and *D*_S‐Se_ under positive *V*_ds_ from 0.00 to 0.40 V. In contrast to the asymmetric counterparts, *D*_S‐S_ and *D*_S‐Se_ display identical *I-V* curves under positive and negative voltage due to the symmetric band diagrams and identical transport mechanisms at the two interfaces. Because the current in *D*_S‐S_ has only a weak dependence on *T* (Figure 2(c)), we speculated that the transmission in *D*_S‐S_ is dominated by direct quantum tunneling. Unlike F-N tunneling, the direct tunneling model predicts *I*_ds_ to vary linearly with *V*_ds_$I\left(V\right)\propto V\exp\left(-2d_{\phi }\sqrt{2m^{*}\phi }/\left.\hbar \right)\right)$ [53, 54]. Above 0.30 V, however, F-N tunneling does occur, as shown in Figure 2(c) and Figure S2. In contrast, *I*_ds_ in *D*_S‐Se_ (Figure 2(d)) has exponential temperature dependence, increasing by a factor of 10 between 50 and 300 K, suggesting thermionic excitation to be the main transport mechanism. Under the same conditions, the current *I*_ds_ in *D*_S‐S_(0.40V, 300K) is 1.16 × 10^−15^A, which is about two orders of magnitude lower than *D*_S‐Se_(3.08 × 10^−13^A(0.40V, 300K)).

The differences in the current and temperature dependence above show that *C*_S‐S_ and *C*_S‐Se_ have markedly different transmission mechanisms and magnitudes and the order of their assembly controls the properties of the resultant device. To understand the physical origins of these differences between the two junctions, we plotted the band structures of *C*_S‐S_ and *C*_S‐Se_ (Figures 2(e) and 2(f)). These band structures show that the metallic properties of 1T MoS_2_ are well preserved in *C*_S‐S_ and *C*_S‐Se_. Further, the band structure of MoSSe is almost unchanged relative to the isolated monolayer band structure (Figure S1). These largely retained band configurations, interlayer distances typical of vdW interactions, and weak adsorption energies clearly suggest that the interactions between the two layers are dominated by the vdW forces. This vdW interface effectively suppresses the FLP effect, due to the reduction of localized densities of states at the interface, metal-induced gap states, and defect/disorder-induced gap states [38, 55, 56].

Schottky barrier heights *ϕ*_n_ and *ϕ*_p_ can be calculated from band structures of the metal-semiconductor junction using the following equations [13, 57]:

(1) $$\begin{array}{c} \phi_{\text{n}}=E_{\text{CBM}}-E_{\text{F}}, \end{array}$$

(2) $$\begin{array}{c} \phi_{\text{P}}=E_{\text{F}}-E_{\text{VBM}}, \end{array}$$

where *E*_F_ is the Fermi level of the junction, while *E*_CBM_ and *E*_VBM_ are the CBM and VBM energies of the 2D semiconductor in contact. According to Eqs. (1) and (2), *C*_S‐S_ is an n-type Schottky contact (*ϕ*_n_ and *ϕ*_p_ are 0.76 and 0.98 eV, respectively) with bands bending downward slightly, while *C*_S‐Se_ is a p-type Schottky contact (*ϕ*_n_ and *ϕ*_p_ are 1.26 and 0.45 eV, respectively) with significant upward bending at the metal-semiconductor interface. Notably, although vdW forces dominate the metal-semiconductor interaction, *ϕ*_n_ and *ϕ*_p_ still deviate from the Schottky-Mott rule [58]:

(3) $$\begin{array}{c} \phi_{\text{n}}^{\prime }=W_{\text{metal}}-\chi , \end{array}$$

where *W*_metal_ and *χ* are the work function and electron affinity of 1T MoS_2_ and Janus MoSSe. From equation (3), the idealized Schottky barrier (*ϕ*_n_′) would be expected to be 0.84 eV.

**Figure 3:**
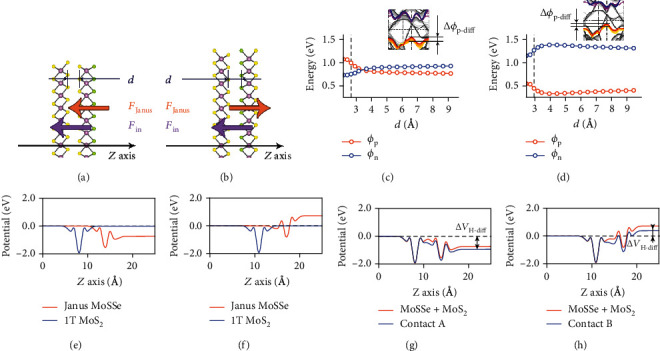
(a), (b) Schematic diagrams of electric field screening effect combined with intrinsic (orange arrows) and interfacial *F* (purple arrows) in contacts with S-S (*C*_S−S_) and S-Se (*C*_S−Se_) interfaces, respectively. The *z* axis perpendicular to the plane is corresponding to the abscissas of subplots (e)-(h). (c), (d) The *ϕ*_p_ and *ϕ*_n_ of *C*_S−S_ and *C*_S−Se_ under different *d*. The vertical grey dashed lines indicate the *d* in balanced states. The illustrations are corresponding band structures of contacts at different *d*. The yellow to red and purple to blue lines indicate the movements of VB and CB of Janus MoSSe. The arrows and tags Δ*ϕ*_p−diff_ indicate the p-type barrier differences. (e), (f) The separated Δ*V*_H−MoSSe_ and Δ*V*_H−MoS2_ of monolayers in *C*_S−S_ and *C*_S−Se_ versus *z* position, respectively. (g), (h) The Δ*V*_H−MoSSe_ + Δ*V*_H−MoS2_ and Δ*V*_H−total_ of *C*_S−S_ and *C*_S−Se_ versus *z* position, respectively. The arrows in plot (g) and (h) indicate the differences of electrostatic potentials from metal to semiconductor (Δ*V*_H−diff_).

The deviation from the ideal Schottky barrier is likely due to the contribution of the inherent field of Janus MoSSe (*F*_Janus_) along with the two interfacial dipoles (*F*_in,S‐S_, *F*_in,S‐Se_). To determine the degree to which each of these elements affects the deviation, we separated the two layers of *C*_s‐s_ and *C*_S‐Se_ and measured the variation of *ϕ*_n_ and *ϕ*_p_ (Figures 3(a) and 3(b)). These calculations show the interface dipole decreases rapidly and that the effect can be ignored at distances larger than 5 Å based on the near-total reversion of the material bands back to their isolated states. To ensure no interaction between systems, the *z* period was set to 150 Å (sensitivity analysis in Figure S3). Interestingly, both *C*_S‐S_ and *C*_S‐Se_ become p-type when *d* > 5 Å and *ϕ*_p_ converge to 0.77 and 0.39 eV, respectively (Figures 3(c) and 3(d)), with the two interfaces being equivalent at 3.28 Å where *C*_S‐S_ transforms from p-type to an n-type structure.

Both *F*_Janus_ and *F*_in_ can contribute to the changes in the Schottky barrier with distance. The magnitude of these effects can be spatially resolved using the Hartree difference potential (Δ*V*_H_) calculated as follows:

(4) $$\begin{array}{c} \Delta V_{\text{H}}=V_{\text{total}}-\sum V_{\text{atom}}, \end{array}$$

where *V*_total_ and *V*_atom_ represent the Hartree potentials of system and each atom. Figures 3(e) and 3(f) show the Δ*V*_H_MoSSeand1TMoS_2_ averaged in the plane along the *z*-axis according to Eq. (4) clearly indicating *F*_Janus_ directs from Se to S atoms (orange arrows in Figures 3(a) and 3(b)) in agreement with previous studies [17]. This results in lower electrostatic potential at the MoSSe side of *C*_S‐S_ and higher potential for the MoSSe side of *C*_S‐Se_, an effect which will naturally perturb the Schottky barrier height. In contrast, Figures 3(g) and 3(h) show the sum of the plane averaged Δ*V*_H_ of the isolated systems (orange lines) as compared to the assembled contacts. In each case, the difference between the isolated and assembled systems corresponds to the effect of *F*_in_.  Figure 3(g) shows *F*_in,S‐S_ to be oriented with *F*_Janus_, pointing from MoSSe to 1T MoS_2_, and increasing the electrostatic potential from metal to semiconductor (Δ*V*_H‐diff_). Inversely, *F*_in,S‐Se_ and *F*_Janus_ have opposite direction, reducing the apparent magnitude of *F*_Janus_ and decreasing Δ*V*_H‐diff_ (Figure 3(h)). These Δ*V*_H‐diff_ calculations are consistent with the deviations from the ideal Schottky barrier and demonstrate how the synergistic effect of *F*_Janus_ and *F*_in_ govern the electronic properties of *C*_S‐S_ and *C*_S‐Se_. The large Δ*V*_H‐diff_ on the sulfur side of MoSSe will cause a greater barrier difference in *C*_S‐S_(Δ*ϕ*_p‐diff_ = 0.31eV) while the smaller Δ*V*_H‐diff_ on the Se side leads to less deviation in *C*_S‐Se_(Δ*ϕ*_p‐diff_ = 0.14eV) This also rationalizes the transition of *C*_S‐S_ from p-type to n-type at *d* ≈ 3.28 Å as the synergistic effect abates.

**Figure 4:**
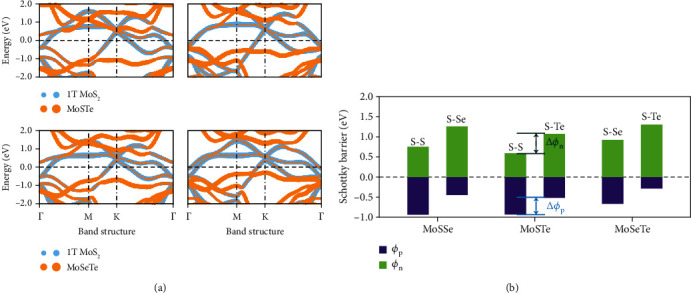
(a) The projected band structures of bilayer 1T MoS_2_-MoSTe with S-S (upper left) and S-Te (upper right) interfaces and 1T MoS_2_-MoSeTe with S-Se (lower left) and S-Te (lower right) interfaces. (b) The asymmetric Schottky barriers of Janus MoSSe, MoSTe, and MoSeTe contacting with metal 1T MoS_2_. The text above each volume indicates the contact interface, where each Janus material has two different contact interfaces. The tags Δ*ϕ*_p_ and Δ*ϕ*_n_ are p-type and n-type Schottky barrier differences between two interfaces.

Finally, to generalize our proposed approach to other 2D Janus materials, we calculated the Schottky barriers in the two sides of Janus MoSTe and MoSeTe contacted with 1T MoS_2_ (Figure S4). Both their projected band structures (Figure 4(a)) and barriers *ϕ*_*p*_ and *ϕ*_*n*_ (Figure 4(b)) indicate these Janus MSMs to also exhibit asymmetric SBHs. Notably, while Janus MoSSe would generate the largest Δ*ϕ*_*p*_ (Δ*ϕ*_*n*_) among these three structures, the relationship is not linear with the vacuum level shifts of the free-standing Janus layers Δ*E*_vac‐MoSTe_ > Δ*E*_vac‐MoSSe_ > Δ*E*_vac‐MoSeTe_ [21]. This initial result shows how the dipole of a Janus structure, the initial work function, and charge transfer also have a great influence on the asymmetry of barriers.

## 4. Conclusions

We demonstrate that 1T MoS_2_ and Janus MoSSe can form an asymmetric van der Waals MSM device with two different barriers contacted back to back through reversing the intrinsic dipole of Janus MoSSe. The transmissions in this MSM device under positive and negative *V*_ds_ are dominated by thermionic excitation and tunneling, respectively, and the rectifying directions and ratios could be effectively controlled by temperature. Furthermore, through the calculations of the differences of electrostatic potentials, we found that the rectifying behaviors hugely associate with the interaction between the intrinsic electric field of the Janus layer and the field induced at the interface. The asymmetric Schottky barrier heights inherent to this design are caused by the intrinsic field of the Janus semiconductor, meaning there is ample room to explore other 2D metals in the search for new MSM devices and the design of “all-2D” flexible high-performance rectifiers.

## Supplementary Materials

Supplementary Materials The atomic structures and band structures of Janus MoSSe and 1T MoS_2_, respectively; the plotted ln (I/*V*^2^) *vs.* ln (1/*V*) and 1/*V* for *D*_S−S_; the *ϕ*_p_ and *ϕ*_n_ movements at *K* point of *C*_S−S_ and *C*_S−Se_; the vdW contact structures of Janus MoSTe (or MoSeTe) and 1T MoS_2_.
