# Supplementary material for: Asymmetric Schottky Contacts in van der Waals Metal-Semiconductor-Metal Structures Based on Two-Dimensional Janus Materials
Source: Research (Wash D C). 2020 Nov 15;2020:6727524. doi: 10.34133/2020/6727524 (PMC7877374; doi:10.34133/2020/6727524)
Supplement: Supplementary Materials — The atomic structures and band structures of Janus MoSSe and 1T MoS2, respectively; the plotted ln (I/V2) vs. ln (1/V) and 1/V for DS−S; the ϕp and ϕn movements at K point of CS−S and CS−Se; the vdW contact structures of Janus MoSTe (or MoSeTe) and 1T MoS2. [file 6727524.f1.docx]

Supporting Information

Asymmetric Schottky Contacts in van der Waals Metal-Semiconductor-Metal Structures Based on Two-dimensional Janus Materials

*Jia Liu,*^1^ *Ji-Chang Ren,*^1^ *Tao Shen,*^1^ *Xinyi Liu,*^1^ *Christopher J. Butch,*^2^ *Shuang Li,*^1^^[[1]](#footnote-1)^* *and Wei Liu*^1*^

^1^ Nano and Heterogeneous Materials Center, School of Materials Science and Engineering, Nanjing University of Science and Technology, Nanjing 210094, China

^2^ Department of Biomedical Engineering, Nanjing University, Nanjing, China; Blue Marble Space Institute of Science, Seattle, Washington


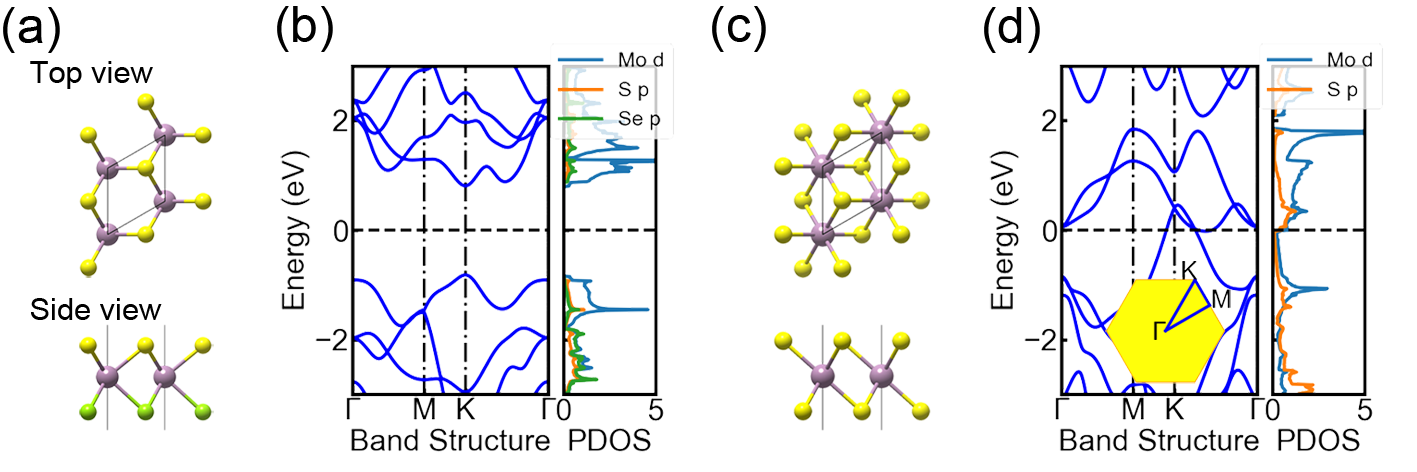


Figure S1. The atomic structures and band structures of (a, b) Janus MoSSe and (c, d) 1T MoS_2_, respectively. The Brillouin zone path is depicted in (d) as an illustration. The Fermi levels of Janus MoSSe and 1T MoS_2_ are referred to zeros in band structures.


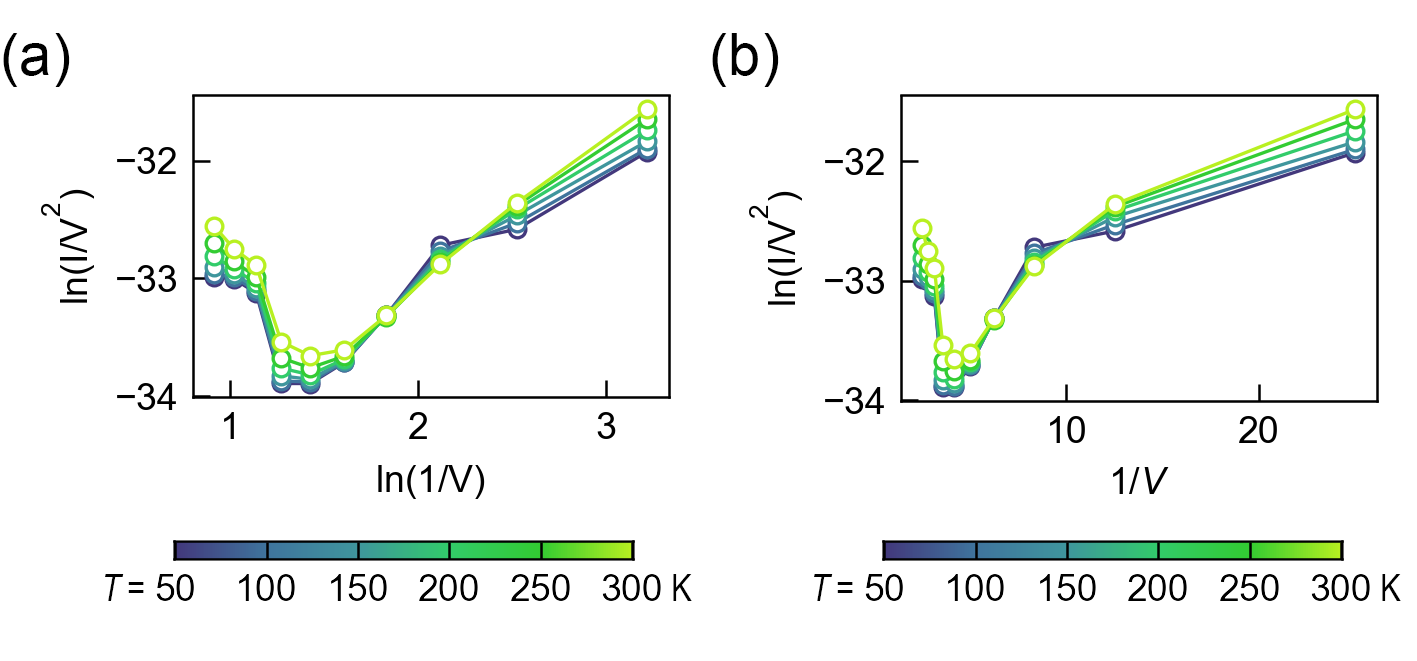


Figure S2. (a) The plotted ln(*I* / *V*^2^) *vs.* (a) ln(1 / *V*) and (b) 1 / *V* for D_S-S_. The equations $I(V)\propto V\exp\left( -\frac{2d_{\phi}\sqrt{2m^{*}\phi}}{\hbar} \right)$ for direct tunneling and $I\left( V \right)\propto V^{2}\exp\left( -\frac{4d_{\phi}\sqrt{2m^{*}\phi^{3}}}{3\hbar qV} \right)$ for F-N tunneling in manuscript could be linearized in logarithm scales, and become $\ln\left( \frac{I}{V^{2}} \right)\propto\ln\left( \frac{1}{V} \right)-\frac{2d_{\phi}\sqrt{2m^{*}\phi}}{\hbar}$ and $\ln\left( \frac{I}{V^{2}} \right)\propto-\frac{1}{V}\left( \frac{4d_{\phi}\sqrt{2m^{*}\phi^{3}}}{3\hbar q} \right)$, where *ħ* is the reduced Planck constant, *m*^*^ is the effective mass of carrier in system, and *d_ϕ_* is barrier width [1-2]. The nearly linear plot of ln(*I* / *V*^2^) *vs.* ln(1 / *V*) with *V*_ds_ < 0.3 V indicates a direct tunneling, while the nearly linear plot of ln(*I* / *V*^2^) *vs.* *1* / *V* with *V*_ds_ > 0.3 V favors an F-N tunneling in D_S-S_.


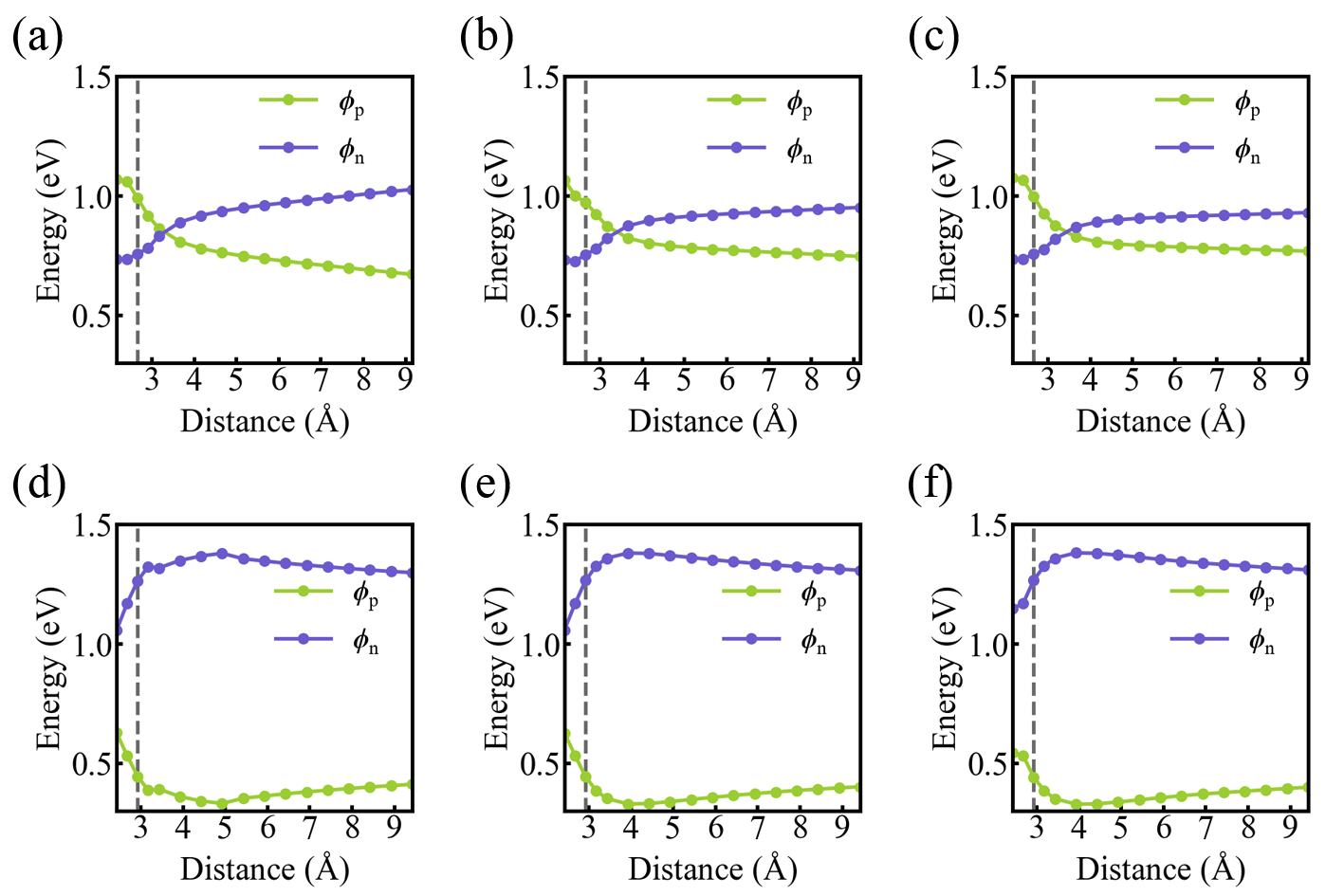


Figure S3. The *ϕ*_p_ and *ϕ*_n_ movements at K point of (a-c) C_S-S_ and (d-f) C_S-Se_ with z axis = 50, 100 and 150 Å. The grey dashed lines indicate the *d* of balanced states. It could be seen that the movements are converged when z axis = 150 Å.


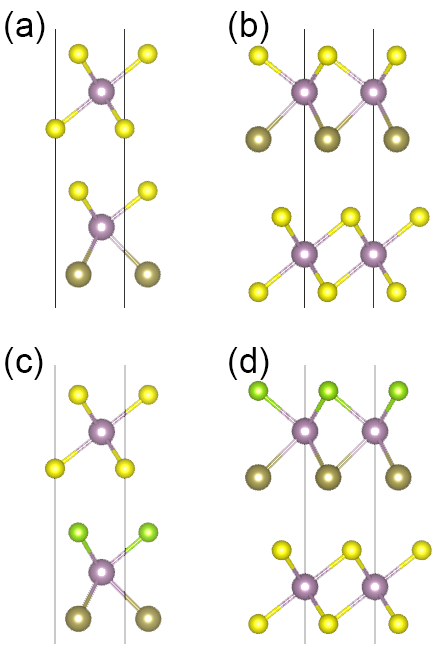


Figure S4. The vdW contact structures of Janus MoSTe (or MoSeTe) and 1T MoS_2_ with (a) S-S, (b) S-Te, (c) S-Se and (d) S-Te interfaces. The violet, yellow, green and brown spheres represent Mo, S, Se and Te atoms, respectively.

**REFERENCES**

[1] F. Ahmed, M. S. Choi, X. Liu and W. J. Yoo, "Carrier transport at the metal–MoS_2_ interface," *Nanoscale*, vol. 7, no. 20, pp. 9222, 2015.

[2] B. K. Sarker and S. I. Khondaker, "Thermionic emission and tunneling at carbon nanotube–organic semiconductor interface," *ACS Nano*, vol. 6, no. 6, pp. 4993, 2012.

1. * Corresponding authors: [lishuang@njust.edu.cn](mailto:lishuang@njust.edu.cn); weiliu@njust.edu.cn [↑](#footnote-ref-1)
